# From neglect to spotlight: the underappreciated role of B cells in cutaneous inflammatory diseases

**DOI:** 10.3389/fimmu.2024.1328785

**Published:** 2024-02-15

**Authors:** Eun-Gang Lee, Ji Eun Oh

**Affiliations:** ^1^ Graduate School of Medical Science and Engineering, Korea Advanced Institute of Science and Technology (KAIST), Daejeon, Republic of Korea; ^2^ BioMedical Research Center, KAIST, Daejeon, Republic of Korea

**Keywords:** B cells, skin, inflammatory disease, pemphigus vulgaris, systemic lupus erythematosus, atopic dermatitis, skin-infiltrating B cells

## Abstract

The skin, covering our entire body as its largest organ, manifests enormous complexities and a profound interplay of systemic and local responses. In this heterogeneous domain, B cells were considered strangers. Yet, recent studies have highlighted their existence in the skin and their distinct role in modulating cutaneous immunity across various immune contexts. Accumulating evidence is progressively shedding light on the significance of B cells in maintaining skin health and in skin disorders. Herein, we integrate current insights on the systemic and local contributions of B cells in three prevalent inflammatory skin conditions: Pemphigus Vulgaris (PV), Systemic Lupus Erythematosus (SLE), and Atopic Dermatitis (AD), underscoring the previously underappreciated importance of B cells within skin immunity. Moreover, we address the potential adverse effects of current treatments used for skin diseases, emphasizing their unintentional consequences on B cells. These comprehensive approaches may pave the way for innovative therapeutic strategies that effectively address the intricate nature of skin disorders.

## 1 Introduction

The skin is a crucial immune component, serving as the first line of defense against numerous threats, encompassing physical and chemical damage as well as infections arising from a variety of pathogens, including bacteria, viruses, fungi, and helminths. To fulfill this protective role, the skin consists of various types of immune cells, each with distinct functions and responsibilities (1). Over the last few decades, significant advances have been made in unraveling the existence and roles of a diverse array of specialized leucocytes that constitute cutaneous immunity, thereby further highlighting crosstalk among these cellular components (2).

In the context of innate immunity, Langerhans cells (LCs) and dermal dendritic cells (dDCs) are the central players with well-characterized roles as antigen-presenting cells (APCs) (3). Concurrently, multiple literatures underscore the significance of T cells in cutaneous immunity. Notably, the skin harbors nearly 5% of all human T cells, a proportion roughly double that found in the peripheral circulation (4, 5). Furthermore, in alignment with the emerging concept of tissue-resident memory T cells (TRMs), the crucial role of skin TRMs has been elucidated that they can generate rapid and localized responses to external stimuli (6). However, the significance of B cells, which constitute the other arm of adaptive immunity, within the cutaneous immune system has not been well-defined.

Recent findings have conferred the presence of B cells in the skin of both humans and animals, compelling a reevaluation of their significance in cutaneous immunity (7–14). These B cells localized within the skin may maintain cutaneous immunity through various mechanisms, including antibody production, cytokine secretion, and antigen presentation. In addition, different and distinct B cell subsets have been characterized in the context of inflammatory dermatoses, present either in the lesional skin or within the systemic circulation. With the evolving insights into skin-associated B cells, it is imperative to distinguish their roles in both homeostasis and disease. Specifically, it is crucial to determine whether these B cells exhibit i) pro- or anti-inflammatory behaviors, ii) protective or pathogenic attributes, and iii) systemic effects or specialized local effects.

In this article, we review recent findings on systemic and skin-localized B cells in inflammatory skin diseases to uncover their underestimated roles in the cutaneous immune system. Additionally, we propose the potential side effects of current therapeutic agents prescribed to patients with cutaneous inflammatory disorders, with a focus on their inadvertent impact on B cells.

## 2 Presence of B cells in the skin

### 2.1 B cells in healthy skin and homeostasis

It was believed that B cells were not present in normal human skin, substantiated by evidence from previously reported histological studies (11, 15). However, these studies inherently had limitations to detect and quantify very few amounts of cells. The advent of advanced methodologies, afferent lymphatic cannulation and flow cytometry, has unveiled the presence of B cells localized within the skin, demonstrated in both humans (11, 16) and animals (12–14). These skin-localized B cells were detected in the dermis, but not detected in the epidermis. They are typically dispersed as isolated cells during homeostasis whereas they tend to form clusters or more structured lymphoid formations during inflammation (14).

Skin B cells exhibit a heterogeneous phenotype, consisting of innate-like B cells that resemble B-1 B cells expressing high levels of IgM and CD11b, as well as conventional B-2 B cells (12–14). Remarkably, the skin of mice exhibits a significant enrichment of innate-like B cells with a substantial proportion of interleukin-10 (IL-10)-producing cells, indicating their potential significance in modulating inflammation within the cutaneous immune system. In accordance with this notion, peritoneal B-1 B cells in mice selectively migrate into the inflamed skin (14). Considering the importance of pattern recognition receptors (PRRs), particularly Toll-like receptors (TLRs), in the development and function of B-1 B cells (17–19), exploring the expression and activity of TLRs on innate-like B cells in the skin presents an exciting research avenue. However, current understanding of PRR expression on skin-localized B cells remains limited, requiring future explorations to unravel the specifics of innate signaling in these cells. For conventional B-2 cells, human IgG^+^ B-2 B cells that infiltrate the skin have fewer IgG1 subclass and tend to use specific Vh genes compared to their counterparts in the blood, indicating the presence of distinct B cell subsets specialized for the skin (20).

Beyond their role in regulating cutaneous inflammation, skin B cells also contribute to the reinforcement of barrier function to prevent infection. Innate-like (B-1) B cells typically recognize conserved structures of pathogens and produce polyreactive natural antibodies even without prior exposure to antigens. In humans, skin-localized plasma cells secret IgA in eccrine sweat glands. These secreted antibodies in sweat bind to pathogenic microbes and skin commensals, regulating their population and activity (21–23). Recently, Wilson et al. (24) demonstrated that healthy human and mouse skin naturally harbors antibody-secreting plasma cells and plasmablasts, predominantly IgM-secreting cells. These cells arose mainly from the B-1 lineage and developed independently of microbiota and T cells, producing natural IgM. Notably, chronic skin inflammation significantly increased the number of these IgM-secreting cells. Collectively, this study suggests a role of skin plasma cells in supporting homeostatic skin functions and offering defense against pathogens.

Skin-localized B cells also enhance other immune functions. Activated B cells in the skin display elevated levels of MHC class II and costimulatory molecules (CD80/86), equipping them aptly for T cell activation (12). Furthermore, a recent study indicates that tissue-resident B cells spatially co-localize with macrophages, influencing their polarization and function, thereby playing a key role in maintaining tissue homeostasis (25). Although skin was not included in the analyzed tissues in this particular study, it implies a pivotal role for tissue-resident B cells in overall immune regulation.

Skin microbiota play a crucial role in managing cutaneous physiological and pathological processes like wound healing and inflammation (26). Similar to the role of antibodies in shaping the gut microbiome, they might also influence the cutaneous microbial communities. Electron microscopy of human skin indicated that skin microbes are enveloped by IgA, IgG, and IgM, suggesting the involvement antibodies in managing microbial colonization in skin (23). This implies that secretory antibodies may affect microbial populations in the skin similarly to their impact in the gut. Further investigations are needed to determine how antibodies can regulate the microbial colonization in different skin areas.

Given its constant exposure to diverse external insults, the skin remains susceptible to a diverse array of threats. Therefore, tissue repair and wound healing are vital properties of the skin. The role of skin-localized B cells in wound healing was demonstrated through studies using B-cell-deficient mice, which exhibited delayed wound healing compared with B-cell-sufficient mice. This indicates a potential role for B cells in facilitating tissue repair (27). Possibly, the innate-like B cells mentioned earlier appear to serve as key players in the wound healing process, as they localize within inflamed skin and regulate inflammation via IL-10 secretion. Taken together, skin-associated B cells emerge as pivotal components not only responsible for enhancing barrier functions but also in maintaining the homeostasis of the cutaneous microenvironment.

### 2.2 Skin-localized B cells: recirculating or resident?

Previous studies have demonstrated that B cells are present in the skin during homeostasis or in disease state. However, only limited aspects of skin-localized B cells started to be unveiled, still leaving a multitude of inquiries unanswered. One of the primary questions is whether they are distinct resident immune cell populations or they transiently migrate into the skin and then recirculate after a certain period.

The concept of tissue-resident memory B cells (BRMs) has emerged with the discovery of memory B cells in the lung for prolonged periods of time after infection (28). Subsequent investigations have confirmed that these B cells are a transcriptionally and functionally distinct population, marked by their residence within tissues (29–32). However, the current understanding of BRMs has been restricted to the lung, leaving their characterization in other barrier tissues uncharted (33). Notably, no BRM formation was shown in the lower female reproductive tract after HSV-2 immunization and subsequent infection (34), implying that the establishment of BRM is highly dependent on tissue microenvironments where localized B cells receive signals for maintenance (33, 34). Hence, whether skin-localized B cells possess the capabilities to evolve into *bona fide* skin BRMs is one of the intriguing questions to extend the frontiers of BRMs.

As skin TRMs have been well-characterized with distinct surface markers and skin-homing factors (35, 36), it is tempting to infer whether skin-localized B cells can also exhibit analogous phenotypes. Cutaneous lymphocyte antigen (CLA) is a well-defined skin-homing receptor for T cells known to engage in ligand-receptor binding with E-selectin on cutaneous vascular endothelium (37, 38). Interestingly, several studies have demonstrated that human activated B cells can also express CLA (39, 40), suggesting a potential role for CLA in mediating B cell migration into the skin as it does in T cells. Furthermore, chemokine receptors like CCR4 and CCR10, found on skin TRMs, are also present on subsets of B cells (41–43). However, these phenotypic similarities are insufficient to conclude that skin-localized B cells are resident population. These markers are homing factors and additional signals that determine the retention of B cells in the skin are still elusive. Thus, further research is needed to ascertain the actual persistence of skin-localized B cells and to elucidate their maintenance mechanisms within the cutaneous microenvironments.

The skin is destined to defend the whole body against a wide range of external threats, thus composed of heterogeneous cellular components. Given this distinctive microenvironment of the skin, it is plausible that B cells could potentially establish residency within the skin following specific exposures, playing a role in mounting a faster and more effective immune response upon subsequent encounters with the same antigen. However, it is also crucial to figure out the potential dual role of these B cells that they could also be involved in the pathophysiology of inflammatory skin diseases by responding to allergens or autoantigens. Indeed, clinical studies in the field of dermatology have reported that recurrent lesions in the same location are strongly related to the tissue-resident populations specific to the disease-associated factors (44–46). Notably, only subsets of T cells have been implicated in driving this recurrent flare, such as autoreactive Th17 cells within fixed psoriatic plaques (6). Although less is known, it is possible that skin-resident B cells might also contribute to the recurring pathophysiology within the skin. Accordingly, a comprehensive investigation into skin-resident B cells should be conducted to distinguish populations that play protective roles from those that harbor pathogenic functions.

## 3 Role of B cells in various inflammatory skin diseases

### 3.1 Integrative profiling of B cells in various inflammatory dermatoses

Although the current understanding of B cells in healthy skin remains limited, their potential involvement and significance in various inflammatory skin diseases are increasingly being elucidated. Conventionally, the role of B cells in cutaneous pathologies has been predominantly recognized as systemic effectors, producing circulating antibodies that enter the skin and trigger inflammation. However, recent emerging evidence has altered this perspective, suggesting that B cells can contribute to the pathogenesis of skin disease not only through systemic mechanisms, but also through local interactions. This is supported by the increased B-cell infiltrates within lesional skin, with a positive correlation observed between the number of skin-infiltrating B cells and disease severity (47–49). These B cells are now considered to exert localized actions, encompassing local antibody production and the formation of tertiary lymphoid organs (TLOs). Therefore, a comprehensive understanding of the systemic and local impact of pathogenic B cells becomes pivotal in developing more precise and efficacious therapeutic strategies for various skin pathologies. In this section, we will delve into an integrative exploration of B-cell profiles in three representative inflammatory dermatoses including pemphigus vulgaris, systemic lupus erythematosus, and atopic dermatitis.

#### 3.1.1 Pemphigus vulgaris

Pemphigus vulgaris (PV) is an autoimmune skin disorder characterized by severe blistering of the skin and mucosa. It is caused by aberrant autoantibodies that target two specific desmosomal proteins, desmoglein-1 (DSG1) and desmoglein-3 (DSG3), which are essential for maintaining epidermal keratinocyte adhesion (50). Remarkably, the disease severity of PV has been correlated with the abundance of serum DSG1/3-specific autoantibody. The well-characterized antigenic specificity for DSG1/3 has allowed researchers to isolate these pathogenic B cells from the peripheral blood of PV patients, thus elucidating their distinctive transcriptomic and phenotypic profiles (51–53). Detection of DSG1/3-specific B cells in PV patients demonstrated that DSG1/3-specific memory B cells also correlate with disease severity (51, 52). Also, transcriptome analysis of autoreactive B cells performed at a single-cell level identified differentially expressed genes, encompassing those associated with T-cell costimulation (*CD137L*), B-cell differentiation (*CD9, BATF, TIMP-1*), and proinflammatory cytokines (*S100A8, S100A9, CCL3*) (51).

**Figure 1:**
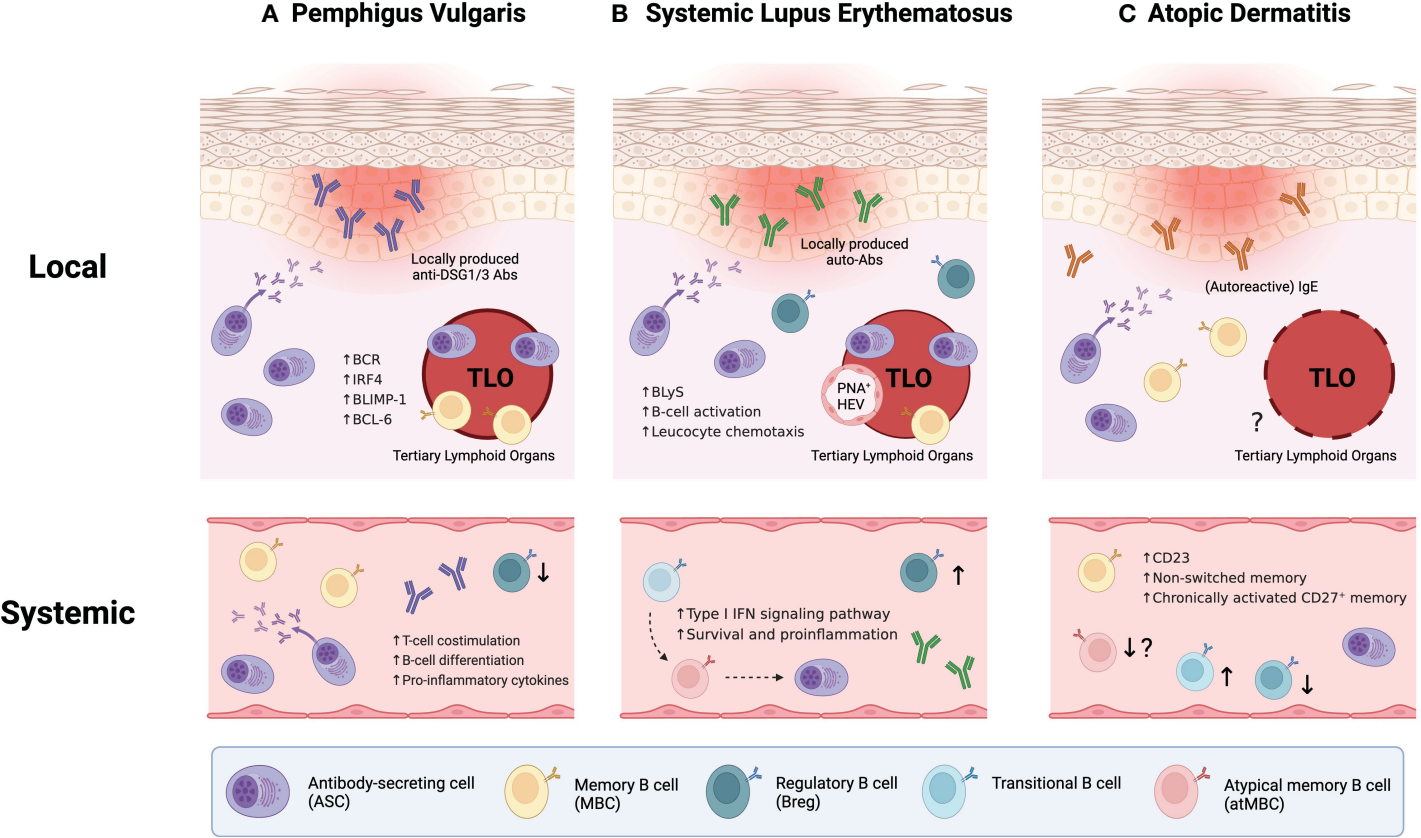
Integrative B-cell profiling in the pathophysiology of inflammatory skin diseases. **(A)** In pemphigus vulgaris (PV), disease severity correlates with both the level of circulating anti-DSG1/3 autoantibodies and the portion of DSG1/3-specific B cells in peripheral blood. Also, skin-localized B cells play crucial roles in disease progression by forming cutaneous tertiary lymphoid organs (TLOs) with IL-21^+^CD4^+^ T helper cells and locally producing anti-DSG1/3 antibodies. **(B)** In systemic lupus erythematosus (SLE), recently identified B cell subpopulations, namely atypical memory B cells (atMBCs) and CD27^-^IgD^-^ double-negative B cells (DN2 B cells), appear poised to differentiate into antibody-secreting cells (ASCs), which may amplify the disease severity (dashed arrows indicate potential differentiation pathways). Similar to PV, local contribution of skin-localized B cells in the pathogenesis of SLE has been elucidated through enhanced B cell infiltration, TLO formation in lesional skin, and presence of distinct germinal centers with peripheral node addressin(+) high endothelial venules (PNA^+^ HEVs). **(C)** Unlike the two previous disorders, the establishment of TLOs has not been demonstrated yet in atopic dermatitis (AD). However, recent AD research suggests the concept of “autoallergy”, where autoimmunity and atopic conditions overlap, marked by autoreactive IgE antibodies. Also, systemic B-cell abnormalities have been observed in the patients with AD.

While circulating anti-DSG1/3 autoantibodies have been considered the primary etiology of PV (54), recent discoveries have underscores the importance of skin-localized B cells in this disorder (**Figure 1A**). These B cells have been characterized by localized antibody production and the formation of cutaneous TLO (47, 55). Comparative analysis demonstrated that DSG1/3-specific B cells and plasma cells were localized to the lesional skin of PV patient, distinct from healthy skin, and these B cells aggregated with IL-21^+^ CD4^+^ T helper cells forming TLOs (47). Further dissection through isolation and *in vitro* culture of cutaneous B cells revealed that they produce larger amounts of anti-DSG1/3 antibodies and enriched B cell receptors (BCRs), compared to their circulating counterparts from the same patients (55). Moreover, B cells present within TLOs expressed high levels of transcription factors associated with B-cell differentiation, including BLIMP-1, IRF4, and BCL-6 (55), indicating that they are a distinctive population with a high propensity to becoming antibody-secreting cells (ASCs). Taken together, skin-localized B cells coupled with TLO formation in PV appear to be primary effectors propelling disease progression, suggesting the potential benefit of *in situ* targeting for therapeutic intervention.

#### 3.1.2 Systemic lupus erythematosus

Systemic lupus erythematosus (SLE) is a multisystem autoimmune disorder that often presents with skin manifestations. It is caused by the disruption in central tolerance, allowing autoreactive lymphocytes to survive and elicit a cascade of abnormalities including the production of various autoantibodies like antinuclear antibodies and anti-dsDNA antibodies (56). Unlike PV, SLE involves heterogeneous populations of autoreactive lymphocytes with distinct antigen specificities. Whereas pathogenic autoantibody is sufficient to drive cutaneous pathology in PV (54), self-reactive autoantibodies alone appear insufficient to induce skin manifestations in SLE (57). Furthermore, not all SLE-associated autoantibodies correlate with disease activities (58), underscoring its complex and heterogeneous immunopathogenesis.

In SLE patients, 25–50% of the mature naïve B cells generate autoantibodies, suggesting defective and abnormal self-tolerance at the early stages of B-cell development (59). This has led researchers to investigate B-cell abnormalities in SLE. Recent exploration has extended to newly defined B-cell subpopulations, such as atypical memory B cells (atMBCs) (60). In peripheral blood from SLE patients, the B-cell compartment exhibited significant dysregulation, featuring elevated proportions of transitional B cells, atMBCs, and ASCs, while unswitched memory B cells showed a reduced proportion (**Figure 1B**). Remarkably, transitional B cells were intensively activated through type I interferon (IFN) signaling pathway with increased survival and promoted proinflammatory properties (61). These autoreactive transitional B cells may differentiate into atMBCs and subsequently give rise to ASCs, particularly through an extrafollicular pathway. This process constructs a potential pathogenesis axis that spans from transitional B cells to ASCs (60). Recently, CD27 and IgD double-negative-2 B cells (DN2 B cells), akin to atMBCs, have been also implicated as the major precursor of ASCs and associated with the pathogenesis in patients with rheumatoid arthritis (RA) (62). Collectively, these parallels between the two prevalent autoimmune diseases potentially converge upon the pathogenic role of atMBCs or DN2 B cells differentiating into autoreactive ASCs. However, it remains to be delineated whether such alteration in the B-cell compartment occurs in other affected organs within SLE patients, encompassing kidneys, joints, and skin.

Beyond the systemic contribution of B cells in driving SLE pathogenesis, local humoral responses in each affected tissue are of particular importance. Notably, some circulating autoantibodies display weak correlation with disease activity in SLE patients (59), postulating that tissue-resident immune cells and locally generated antibodies play a crucial role in disease progression within specific tissues. Like the aforementioned research in PV, the manifestation of cutaneous lupus involves enhanced B cell infiltration and TLO formation in affected skin (63–66). These skin-localized B cells organize into aggregates coordinated with CD3^+^ T cells (64, 65), suggesting their additional roles such as cytokine secretion and antigen presentation to pathogenic T cells beyond their autoantibody production. Dissecting the TLOs revealed that it contains distinct germinal center (GC) structures complemented by auxiliary components like peripheral node addressin(+) high endothelial venules (PNA^+^ HEVs) and CXCL13^+^ cells (66). Autoreactive B cells in cutaneous lesions are transcriptionally distinct compared to those in healthy controls. These B cells exhibit gene signatures enriched in B cell activation and leucocyte chemotaxis pathways (67). Also, B lymphocyte stimulator (BLyS), a pivotal factor for B cell activation and survival, was significantly upregulated in lesional skin (68), supporting enhanced B cell activation in the context of cutaneous lupus pathologies. Taken together, recent findings in cutaneous lupus have demonstrated the considerable contribution of skin-localized autoreactive B cells to the pathophysiology of SLE at the tissue level. Consequently, they highlight the need to integrate altered B-cell phenotypes in the peripheral circulation and the actual effector mechanisms at the site of lesions.

#### 3.1.3 Atopic dermatitis

Atopic dermatitis (AD) is one of the most common allergic diseases characterized by elevated type 2 immune response and high levels of serum IgE. While the definitive etiology of AD remains incompletely elucidated, a diverse array of heterogeneous factors, spanning from skin barrier disruption, genetic susceptibility, and environmental triggers, converge to shape the pathogenesis of AD (69). Accompanied by intense pruritus, AD prompts scratching on affected skin regions, perpetuating a vicious cycle that exacerbate disease progression.

Intriguingly, recent advances in AD research have reshaped the conventional understanding of AD pathophysiology (70–74). Among these, a notable insight is the emerging concept of “autoallergy”, whereby autoimmunity coexists with atopic conditions, with antigen-specific IgE serving as a prominent hallmark (70, 71). The main driver of autoallergy is IgE-reactive autoallergens that share cross-reactivity and molecular mimicry with exogenous allergens. When keratinocytes are damaged by external stimuli like scratching and environmental factors, they release intracellular autoantigens. These autoantigens might sensitize autoreactive immune cells that exhibit cross-reactivity with exogenous allergens (71). Subsequently, these immune cells experience reactivation upon encountering the cross-reactive exogenous allergen. Although the role of autoreactive T cells in AD has been discussed elsewhere (72), the significance and involvement of B cells in promoting autoallergy in AD patients remains poorly investigated.

Also, dramatic advances in multi-omics analyses have made it possible to classify AD subtypes and endotypes (73, 74). This classification considers age (pediatric and adult), specific IgE to allergens (extrinsic and intrinsic), and presiding immune response (acute and chronic) (73). While extrinsic AD exhibits conventional AD traits with high serum IgE, intrinsic AD shows normal serum IgE levels, implying different contributions of pathogenic B cells to the pathology of each AD endotype. Hence, the characterization of B cells in AD patients is of particular importance for further refining AD categorization and subsequently developing precision therapy for individual AD endotypes.

B cells have been implicated in perpetuating chronic inflammation in AD pathophysiology. Experimental induction of AD in animal models using CD19-deficient mice demonstrated a significant reduction in disease severity and attenuated activities of CD4^+^ T cells compared to wild-type mice (75). Intriguingly, when wild-type B cells were adoptively transferred into CD19-deficient mice, the disease severity was restored to levels observed in wild-type mice. Therefore, it is likely that subsets of CD19^+^ B cells play a pathogenic role in AD pathophysiology, although their precise involvement has not been fully elucidated.

A recent study demonstrated local and systemic B-cell abnormalities in patients with AD (49) (**Figure 1C**). The authors examined B cell subsets in the peripheral blood and skin of patients with AD or psoriasis, comparing them to healthy controls. Strikingly, overall B-cell frequencies were higher in both skin and blood of AD patients, and these increased B cells also exhibited elevated CD23 expression compared to the other two groups. CD23 expression on B cells was also correlated with the severity of AD, suggesting a potential role of CD23 in AD pathology. Previous studies have proposed CD23 as a predictive marker for B cell fate decision; CD23^+^ B cells tend to remain in the GCs whereas CD23^-^ B cells preferentially differentiate into ASCs (76–78). Moreover, CD23 expression is induced by IL-4 signaling, which closely correlates with hyperactivated type 2 immunity characterized in allergic diseases (79). Therefore, this IL-4/CD23 interplay might be attributed to the dysregulation and dysfunction of B-cell subpopulations, ultimately triggering the pathophysiology of AD. The authors also presented alterations in other B-cell subsets; aforementioned CD27^-^IgD^-^ DN2 B-cell counts were lower whereas transitional B-cell counts were higher in the blood of AD patients. However, B-cell profiling in AD remains less explored compared to the two aforementioned autoimmune diseases, PV and SLE. Consequently, further investigations are needed to characterize the distinct subsets of B cells and their function in the pathogenesis of AD.

In summary, B cells can exert both systemic and localized effects on inflammatory skin conditions such as PV, SLE, and AD (**Figure 1**). In PV and SLE, skin-localized B cells contribute to disease progression by forming TLOs and engaging in local autoantibody secretion and antigen presentation to autoreactive T cells. While TLO formation has not been demonstrated yet in AD contrary to the two dermatoses, systemic aberrancies in B-cell populations have been observed across all three diseases. Therefore, a comprehensive understanding of the role of B cells in each cutaneous pathology is essential to identify and selectively target the pathogenic subpopulations. In addition to conventional phenotyping approaches, spatial transcriptomics and proteomics analyses would be more insightful to elucidate the properties of cutaneous B cells that function within the lesion microenvironment.

### 3.2 Emerging concept of regulatory B cells in cutaneous immunity

It should not be overlooked that not all B cells promote cutaneous inflammation. Some B cells have also been featured with their immunoregulatory functions in skin disease conditions or skin surveillance, later termed “regulatory B cells (Bregs) (80, 81). Although definitive markers for Bregs have not been established, they are mainly characterized by their immunosuppressive role mediated by IL-10 secretion (82). In humans, IL-10^+^ B cells are notably enriched in the transitional B cell subsets characterized by CD24^hi^CD38^hi^, thus suggesting that transitional B cells encompass a broader concept of regulatory B cells (83, 84).

The significance of Bregs in cutaneous inflammatory disorders has been proposed by differential responses observed in patients with different skin diseases receiving B cell depleting therapy such as rituximab (an anti-CD20 monoclonal antibody) (85–87). While B cell depletion contributes to the amelioration of cutaneous inflammation in cases of AD (85), and PV (86), it might conversely induce inflammation in psoriasis (87). It has been postulated that B cell depletion appears to exhibit beneficial effects in antibody-mediated pathologies, yet it could worsen certain diseases where the pathogenic role of B cells remains unclear. In addition, individuals with systemic autoimmune disorders like SLE and RA were reported to develop psoriasis-like inflammation following rituximab infusion (88, 89). Therefore, these contrasting outcomes underscore the presence of distinct B cells with suppressive roles.

During the last two decades, significant progress has been made in understanding the role of Bregs within the context of skin diseases (90). Remarkably, in peripheral blood of patients with SLE, the frequencies of IL-10-producing B cells were significantly higher compared to healthy individuals (84, 91). Not only were these B cells increased in number, some of them localized to the inflamed skin (91). However, several investigations have proposed that Bregs in individuals with SLE display reduced functionality and suppressive capacity compared to those from healthy controls, attributed to their low responsiveness to CD40 stimulation and thus diminished IL-10 production (83).

In contrast to the increased frequencies of IL-10^+^ B cells in SLE, PV is associated with reduced frequencies of circulating IL-10^+^ B cells (92, 93). Interestingly, during the course of disease remission, there was a notable increase in both transitional B cells and IL-10-producing B cells between the patients achieving complete remission and those with incomplete remission, particularly noticeable in the 6 to 9 months after rituximab treatment (94). Like the case in SLE, Bregs in PV also exhibited impaired functionality with a lower capacity to produce IL-10 (95).

The significance of Bregs in AD has been investigated in both human and mouse models (96–98). In AD-like mouse model, CD5^+^CD19^+^CD1d^hi^ regulatory B cell subset (B10 cells) from the AD mice demonstrated impaired functionality in suppressing IgE secretion when compared to the control group (96). The number of IL-10-producing B cells in AD patients also decreased, displaying a negative correlation with disease severity; individuals with severe AD had lower Breg counts than those with mild symptoms (97). A recent study has further supported this inverse correlation between IL-10^+^ Bregs and disease activity (98). The authors reported that the children with extrinsic AD had significantly fewer Bregs than age-matched healthy controls. Importantly, the frequencies of Bregs not only correlated with disease activity but also with circulating follicular T helper (Tfh) cells, which could contribute to the progression of AD. These Bregs also showed impaired suppressive abilities to inhibit differentiation of Tfh cells. Taken together, uncontrollable allergic inflammation in AD pathology is attributed to the impaired function of IL-10-producing Bregs, resulting in promoting disease severity.

In addition to the three disorders previously mentioned, Matsushita et al. highlighted the significance of Bregs in systemic sclerosis (SSc), an autoimmune disease characterized by fibrosis in the skin and other internal organs (99, 100). A clinical study revealed that patients with SSc had fewer IL-10^+^ B cells and CD24^hi^CD27^+^ regulatory B cells in their blood compared to healthy individuals, with a notable inverse correlation between the frequency of Bregs and autoantibody levels (99). Additionally, in a mouse scleroderma model, IL-6^+^ effector B cells exacerbate the disease, while IL-10^+^ regulatory B cells provide protection (100). Moreover, IL-10^+^ Bregs found in the skin play a crucial role in mitigating inflammation and fibrosis in scleroderma, both in skin and lymphoid tissues. Notably, this study suggests that therapeutic strategies targeting B cell activating factor (BAFF) to adjust the balance between effector B cells and regulatory B cells could offer a new direction for treating SSc.

In summary, the collective evidence underscores the suppressive role of IL-10-producing regulatory B cells in mitigating cutaneous inflammation, corroborated by the dysfunctional behavior of Bregs in skin disorders. However, comprehensive understanding of the role of Bregs in skin diseases encounters certain limitations. Most studies on Bregs associated with skin diseases have been primarily performed *in vitro* and different groups adopted different combination of stimuli including CD40L, LPS, CpG, and PMA with ionomycin to induce IL-10 secretion by Bregs (90). These discrepancies in approaches may result in conflicting characterizations of Breg subsets across different research groups, even when focusing on the same disease (83, 91). Furthermore, the majority of studies have concentrated on the IL-10-dependent regulatory function, leaving questions unanswered regarding the IL-10-independent role of Bregs in skin diseases. Accordingly, the populations of representative Breg subsets—IL-10-producing B cells and CD24^hi^CD38^hi^ transitional B cells—do not always exhibit a consistent correlation with each other in various skin disorders (83, 95). Therefore, further systematic investigations are needed to address these limitations and dissect the significance of regulatory B cells in skin diseases.

## 4 Current therapeutics for inflammatory skin diseases and potential side effects on B cells

### 4.1 Current B-cell-directed therapies for cutaneous inflammatory disorders

**Figure 2:**
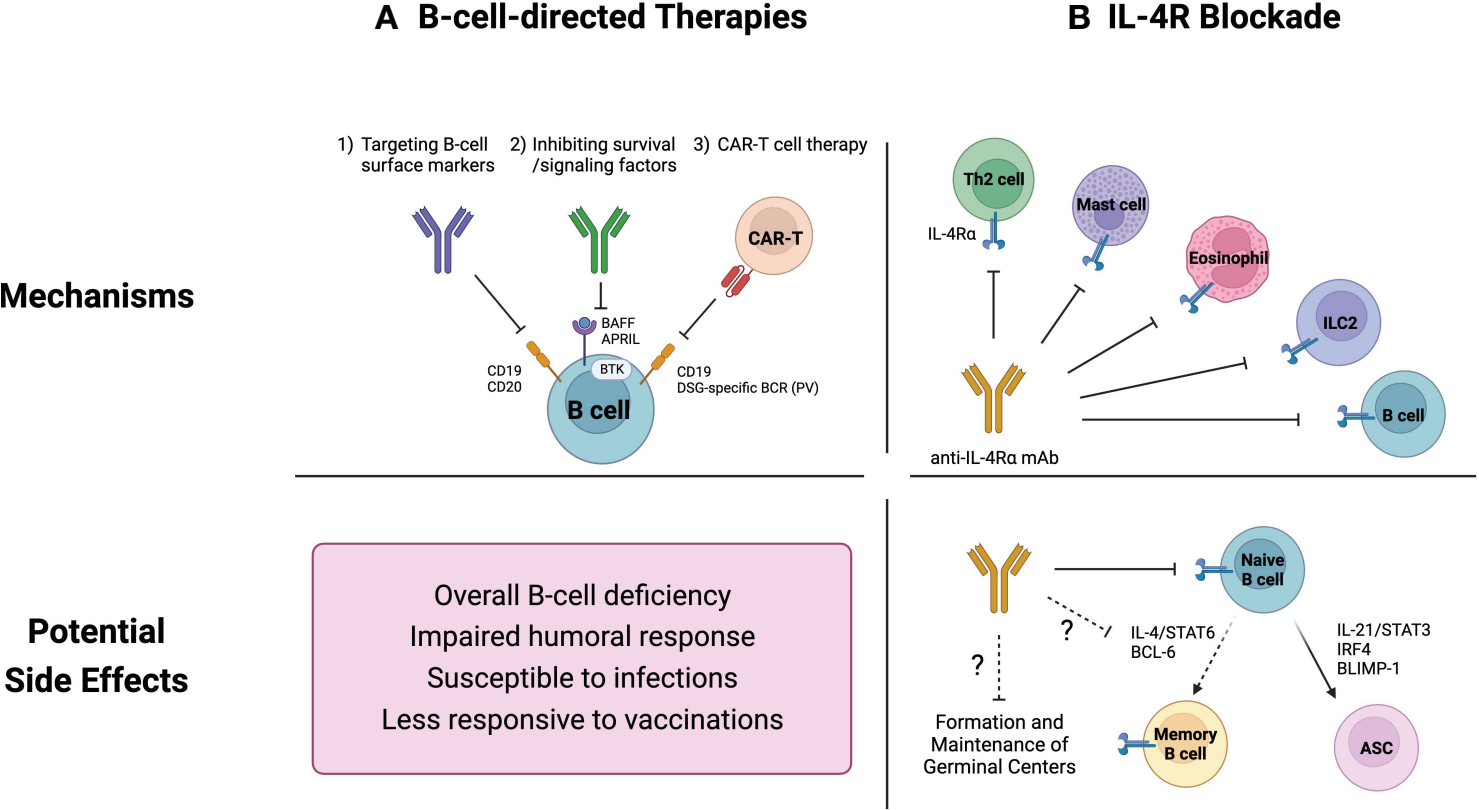
Current therapeutics for inflammatory skin diseases and their potential side effects on B cells. **(A)** B-cell-directed therapies include 1) direct B-cell depletion using monoclonal antibodies that target B cell surface markers, 2) inhibition of survival and signaling factors such as B cell activating factor (BAFF) and Bruton tyrosine kinase (BTK), and 3) chimeric antigen receptor (CAR) T cell therapy. These strategies are not capable of distinguishing between pathogenic and non-pathogenic B cells, resulting in an overall B-cell depletion. This widespread B-cell deficiency may compromise humoral immune responses, leading to increased vulnerability to infections and diminished vaccine efficacy. **(B)** While IL-4R blockade was not initially intended to specifically target B cells, it has emerged as a potential regulator of B cell responses. Naïve B cells rely on IL-4 signaling pathways for their survival and development. Additionally, recent studies have demonstrated the pivotal roles of IL-4 in modulating the germinal center microenvironment. Consequently, IL-4R blockade could potentially suppress overall humoral immunity.

Although the definitive role of B cells in inflammatory skin diseases remains unclear, B-cell-directed therapeutic approaches have demonstrated efficacy in addressing cutaneous pathologies, particularly those involving autoreactive B cells like SLE and PV (101, 102). Current therapeutic strategies aimed at modulating B-cell function can be broadly categorized into three groups; (i) direct depletion of B cells targeting well-defined B cell markers such as CD19, CD20, and CD22, (ii) inhibition of survival and signaling factors, and (iii) chimeric antigen receptor (CAR) T cell therapy (**Figure 2A**).

#### 4.1.1 Direct depletion of B cells targeting B cell markers

Monoclonal antibodies (mAbs) directed against B cell markers like CD19 and CD20 are conventional strategies for targeting B cells, some of which were approved by the FDA (102). An example is rituximab, a chimeric anti-CD20 antibody that has found widespread clinical application across various inflammatory and autoimmune diseases (85–89, 94). It mediates B cell depletion through antibody-dependent cellular cytotoxicity (ADCC) as well as through other B-cell lysis mechanisms including complement-mediated and Fc receptor mediated responses (101, 103).

Rituximab exhibits remarkable efficacy, leading to a significant reduction in pathogenic anti-DSG antibody titers in treated PV patients (104). The pathogenic role of skin-localized B cells in PV has sparked interest in local therapeutic approaches. For example, recent studies showed that direct intralesional injection of reduced-dose rituximab exhibited notable clinical improvement (105, 106). The application of rituximab extends to systemic autoimmune diseases like RA and SLE. While it mediates clinical remission in RA (107, 108), its effects vary for SLE (109, 110). Although rituximab could effectively remove peripheral B cells, autoreactive B cells in the tissues appear to be less affected by systemic B cell depletion (111). Also, rituximab has improved AD symptoms, negatively correlating with total and lesional skin B cell counts (85, 112).

#### 4.1.2 Inhibition of survival and signaling factors

Blocking survival signals and growth factors essential for B cells is another therapeutic intervention for cutaneous inflammatory diseases (101, 102). The major targets of B cell signaling include B cell activating factor/a proliferation-inducing ligand (BAFF/APRIL) and Bruton tyrosine kinase (BTK).

BAFF, a member of the tumor necrosis factor (TNF) superfamily (also known as TNFSF13B), functions as an essential survival cytokine for both B cells and ASCs (113). Its homologous factor, APRIL (also known as TNFSF13) shares similar functions, and therapies targeting BAFF typically inhibit the activity of APRIL (102). Among BAFF inhibitors, belimumab, a fully humanized anti-BAFF mAb stands out and has been approved for SLE treatment (102, 114). Despite elevated BAFF expression in some SLE cases (115, 116), BAFF inhibition with belimumab has shown somewhat variable outcomes among individuals. While it proves efficacious for recalcitrant cutaneous lupus (117), a recent study proposed that substantial proportion of SLE patients do not respond to belimumab (118). Ianalumab (VAY736), a novel BAFF inhibitor targeting BAFF receptor, has attracted great attention as a promising therapeutic agent for SLE and Sjögren syndrome (119). Notably, phase 2 clinical trials are in progress to evaluate its clinical impacts on patients with PV (120).

BTK, belonging to the Tec family of kinases, is a tyrosine kinase expressed across B cell development stages from pre-B to mature B cells, and in myeloid cells as well (121). BTK plays a crucial role in B cell receptor (BCR) activation and both BCR and Fc receptor engagement induce its kinase activity (102). Although usage of BTK inhibitor has predominantly focused on multiple sclerosis (102), its potential for cutaneous inflammatory diseases has been also investigated (120). Remarkably, BTK inhibitors like ibrutinib and rillzabrutinib have shown significant improvement in disease activity in PV patients (10, 122, 123). Rillzabrutinib (PRN1008) is a potent BTK inhibitor known for its distinctive reversible covalent binding, potentially enhancing safety compared to irreversible counterparts like ibrutinib (10). Rillzabrutinib impedes B cell activation by inhibiting BCR, however does not induce B-cell lysis, notably distinct from therapies like rituximab that directly deplete B cells (123).

In addition to the two primary targets, the blockade of CD40 pathway presents a potential target for autoimmune disease intervention due to its critical role in B-cell activation (124, 125). B-cell and T-cell interactions within the GC hinge on the essential costimulatory CD40/CD40L signaling (126). Disruption of this pathway impedes B-cell activation. Interestingly, rituximab has been reported to reduce CD40 expression on B cells (127) and CD40L expression on CD4^+^ T cells (128), thereby mitigating the impact of B-cell-mediated disorders. Consequently, agents that directly target the CD40/CD40L interaction have been developed for more effective inhibition of autoimmune response. Although initial therapies faced significant challenges, with the first anti-CD40L antibody, rupilzumab, being associated with thromboembolic events (129), recent advances have shown promise in reducing these adverse effects (130, 131). Ongoing research is dedicated to creating safer therapeutic variants for autoimmune conditions (125).

#### 4.1.3 Chimeric antigen receptor T cell therapy

CAR T cell therapy is an advanced therapeutic strategy that provides targeted elimination of pathogenic B cells, distinct from the earlier therapeutic methods (102). CD19-targeting CAR T cells have been credited to effectively clearing pathogenic B cells in autoimmune diseases and hematologic malignancies (132, 133). Initially, CAR T cells primarily focused on CD19, lacking selectivity for pathogenic B cell depletion. However, innovative chimeric antigen receptor modifications have enabled precise treatment for autoimmune conditions, exemplified in a PV mouse model (134). The authors engineered CAR T cells expressing the autoantigen Dsg3, forming chimeric autoantibody receptor (CAAR) T cells. These CAAR T cells effectively eliminated pathogenic B cells carrying anti-Dsg3 BCRs without off-target toxicity, thus preserving protective immunity. This approach has extended to human PV, demonstrating its promising preclinical results (135). T cells from PV patients were genetically engineered to incorporate Dsg3 as a decoy receptor on their surface, allowing selective targeting and elimination of B cells producing anti-Dsg3 antibodies. These engineered CAAR T cells effectively cleared patient-derived anti-Dsg3 B cells in experimental cell culture, while sparing non-pathogenic ones.

Promising preclinical results have led to clinical trials for CAR T cell therapies for various diseases (102, 120). One of the remarkable examples is CAR T cells targeting membrane IgE for severe allergic diseases (136). Leveraging their memory properties, these CAR T cells provide prolonged IgE suppression, surpassing the constraints of monoclonal antibodies. Taken together, CAR T cell therapies are broadening its boundaries into autoimmune and allergic disorders, offering targeted depletion and sustained control of pathogenic B cells.

### 4.2 Potential side effects of current therapeutics on B cells

#### 4.2.1 Potential side effects of B-cell-directed therapies

B cell depletion therapies can have diverse effects on B cells and the immune system. While the B-cell-directed therapies effectively target and reduce pathogenic B cells, they can also lead to various side effects (**Figure 2A**).

The primary role of humoral immunity is to protect the body against a wide range of pathogens. The depletion of B cells results in reduced antibody production and impaired defense against pathogens, making individuals more susceptible to infections (137, 138). Compromised humoral immunity may lead to diminished responsiveness to vaccinations, characterized by lower antibody production and impaired memory response upon re-challenge (139–142). Of note, it becomes worth noting that ensuring an effective humoral immune response following vaccination is crucial, as highlighted during the COVID-19 pandemic. Individuals with systemic autoimmune conditions such as multiple sclerosis receiving rituximab treatment exhibited a heightened risk of severe COVID-19 due to impaired humoral immunity. Multiple studies have underscored decreased vaccine response in patients with B cell depletion therapies following SARS-CoV-2 vaccination (139, 141, 142).

Notably, the depletion of B cells can disrupt their maturation and activation process, as evidenced by prolonged rituximab treatment in MS patients, where reappearing B cells exhibited immature yet highly activated characteristics (143). This phenomenon could result in distinct reconstitution patterns of B cell subsets, potentially leading to dysregulation within the B cell compartments. Moreover, B cell depletion therapies seem to have limited impact on plasma cells, as these cells tend to be refractory to such depleting treatments (144). Of particular, long-lived plasma cells residing in the bone marrow demonstrate a significant resistance to B-cell targeting strategies, exhibiting limited susceptibility to depletion (145). The adverse events observed in RA and SLE patients developing psoriatic inflammation post-rituximab treatment could result from these differential reconstitution dynamics within B cell compartments (88, 89). Therefore, a comprehensive understanding of the short-term and long-term effects of B cell depletion on the immune system is needed for optimizing therapeutic strategies and minimizing potential adverse events.

#### 4.2.2 Potential side effects of IL-4Rα blockade on B cells

While B-cell-directed therapies inherently impact B cells, it is intriguing that the interleukin-4 receptor (IL-4R) blockade, initially not designed to specifically target B cells, has emerged as a potential regulator of B cell responses and humoral immunity (76, 146–149) (**Figure 2B**).

Initially identified as B cell growth factor, IL-4 plays a role in promoting the survival and development of IL-4R^+^CD23^+^ naïve B cells (150). It stimulates B cells while protecting them from apoptosis, and enhance their activation, leading to increased expression of activating markers like CD80, CD83, and MHC II, while downregulating inhibitory markers such as CD22 and PD-L1 (146, 151, 152). Furthermore, IL-4 negatively regulates differentiation into ASC, favoring memory B cell (MBC) differentiation pathway (153, 154). Accordingly, the downregulation of IL-4R and CD23 in human naïve B cells primes them for development into activated B cells and plasma cells (77, 78). Notably, CD23^-^ B cells exhibited an ASC-associated gene signature with lower responsiveness to IL-4 signaling, whereas CD23^+^ B cells were associated with an activated gene signature (77).

Recently, the emerging role of IL-4 in modulating the GC microenvironment has gained attention. New insights highlight that not only T helper 2 cells but also T follicular helper cells can produce IL-4, thereby promoting humoral responses and facilitating the GC reaction (155, 156). Unexpectedly, NKT cells also generate an early wave of IL-4 shortly after influenza infection, reaching its peak on the third day post-infection and accounting for a substantial 70% of IL-4-producing cells in the lymph node (147). Furthermore, in the context of antiviral response, IL-4 has been shown to enhance the breadth of the IgG antibody response by expanding rare GC B cells recognizing the shared epitopes (148). Taken together, IL-4 contributes to a rapid humoral response and robust GC reaction, deriving from spatially and temporally distinct sources within the GC.

However, debates have arisen regarding the comprehension of the role of IL-4 in governing humoral responses within the GC microenvironment. Classically, IL-4 has been considered to promote MBC differentiation pathway. Nonetheless, Duan et al. (76) proposed an alternative perspective, suggesting that excess IL-4 availability in the GC might actually suppress MBC differentiation. This assumption allows activated B cells to remain within the GC, preventing them from exiting GC and becoming MBC. To bridge the gap between these conflicting conclusions on whether IL-4 promotes or inhibit MBC differentiation, a recent study has proposed a plausible explanation (149). They demonstrated that IL-4 can prompt GC B cell selection and exit through differential regulation of BCL6 expression, depending on the preexisting levels of BCL6 in a given cell. IL-4 is capable of inducing the negative autoregulation of BCL6, thereby promoting the exit of GC B cells via BCL6 downregulation. Consequently, an excess availability in IL-4 enhanced the exit of GC B cells. However, in the absence of concomitant survival signals, this process can lead to in cell death and a subsequent reduction in the MBC population consistent with the findings of Duan et al. (76). Collectively, IL-4 exhibits an intricate management of the GC microenvironment, manifesting its differential effects and temporal regulation within the GC.

In line with the emerging role of IL-4 in GC regulation, IL-4R blockade might affect humoral immune responses (**Figure 2B**). Dupilumab, an anti-IL-4Rα monoclonal antibody, blocks signaling of major type 2 cytokines, IL-4 and IL-13, both mediated through IL-4Rα chain (157, 158). This biologic agent was FDA-approved in 2017 for use in atopic dermatitis and has shown efficacy in managing AD-related comorbidities such as asthma and allergic rhinitis.

While a 16 to 52-week phase 3 clinical trial of dupilumab did not indicate elevated infectious disease occurrence (159–161), the potential prolonged suppressive influence of IL-4R on maintaining naive B cell homeostasis and proper vaccination response remains uncertain. Indeed, the most common adverse effects of dupilumab, as reported by the U.S. FDA and other institutions, encompass conjunctivitis, upper respiratory tract infections, and skin infections (162). This underscores the potential susceptibility and complications resulting from disruptions in humoral immunity due to impaired IL-4 signaling, eventually making individuals more susceptible to various external threats.

Although less is known about the long-term impact of IL-4R blockade on the immune system, a recent investigation suggested that IL-4R blockade may suppress the development of both naïve and memory B cells (163). They examined the B-cell transcriptome and closely monitored the response to the COVID-19 vaccine in a patient with AD undergoing dupilumab treatment. Transcriptomic analysis revealed reduced frequencies of *IL4R^+^IGHD^+^* naïve B cells and downregulation of *IL4R, FCER2* (CD23), and *IGHD* in the patient treated with dupilumab. The dupilumab therapy resulted in an increased expression of genes linked to apoptosis and suppression of BCR signaling, coupled with the decreased expression of genes associated with class-switching and memory B cell development. Remarkably, after receiving dupilumab, the patient showed a rapid decline in antibodies targeting the SARS-CoV-2 spike protein and receptor binding domain within 4 to 11 months following vaccination (163). Taken together, these findings highlight the critical role of intact and persistent IL-4 signaling in sustaining robust survival and development of naïve B cells, as well as in supporting a prolonged vaccine response.

## 5 Concluding remarks

Traditionally, B cells were not regarded as central actors in the skin immune system. Recent discoveries, however, have prompted a reevaluation on their significance in cutaneous immunity. In this review, we explored the comprehensive involvement of B cells across diverse cutaneous immune contexts, encompassing skin homeostasis, inflammatory conditions, and therapeutic interventions.

Although the focus has primarily been on their systemic roles, such as circulating antibody production, their local actions within the cutaneous microenvironment warrant deeper investigation, particularly their migration and residency mechanisms. Moreover, mapping the presence and functions of skin-resident B cells across various cutaneous immune landscapes remains a priority. Understanding how B cells interact with other cutaneous immune components will illuminate their roles in the larger immune network, potentially revealing novel intervention points for inflammatory skin diseases. An in-depth characterization of the different B cell subsets is crucial for discerning their protective or pathogenic roles within specific diseases. Additionally, the broader impact of B cell alterations on humoral immunity in each skin disorder needs further investigation, including the prolonged implications of cutaneous disease treatment on B cell function and population dynamics.

In conclusion, B cells emerge as multifaceted contributors to cutaneous immunity. A comprehensive investigation of their roles is expected to reveal new dimensions in skin pathophysiology. Through these endeavors, we anticipate to develop innovative treatment modalities, addressing the complexity and heterogeneity inherent in skin diseases.

## Author contributions

EL: Conceptualization, Investigation, Writing – original draft. JO: Conceptualization, Supervision, Writing – review & editing.

## References

[1] Pasparakis M Haase I Nestle FO. Mechanisms regulating skin immunity and inflammation. Nat Rev Immunol (2014) 14:289–301. doi: 10.1038/nri3646 24722477

[2] Ho AW Kupper TS. T cells and the skin: from protective immunity to inflammatory skin disorders. Nat Rev Immunol (2019) 19:490–502. doi: 10.1038/s41577-019-0162-3 30992525

[3] Malissen B Tamoutounour S Henri S. The origins and functions of dendritic cells and macrophages in the skin. Nat Rev Immunol (2014) 14:417–28. doi: 10.1038/nri3683 24854591

[4] Clark RA Chong B Mirchandani N Brinster NK Yamanaka K Dowgiert RK. The vast majority of cla+ T cells are resident in normal skin. J Immunol (2006) 176:4431–9. doi: 10.4049/jimmunol.176.7.4431 16547281

[5] Farber DL Yudanin NA Restifo NP. Human memory T cells: generation, compartmentalization and homeostasis. Nat Rev Immunol (2014) 14:24–35. doi: 10.1038/nri3567 24336101 PMC4032067

[6] Clark RA. Skin-resident T cells: the ups and downs of on site immunity. J Invest Dermatol (2010) 130:362–70. doi: 10.1038/jid.2009.247 PMC2922675 19675575

[7] Egbuniwe IU Karagiannis SN Nestle FO Lacy KE. Revisiting the role of B cells in skin immune surveillance. Trends Immunol (2015) 36:102–11. doi: 10.1016/j.it.2014.12.006 25616715

[8] Debes GF McGettigan SE. Skin-associated B cells in health and inflammation. J Immunol (2019) 202:1659–66. doi: 10.4049/jimmunol.1801211 PMC6402607 30833422

[9] Lerman I Mitchell DC Richardson CT. Human cutaneous B cells: what do we really know? Ann Transl Med (2021) 9:440. doi: 10.21037/atm-20-5185 33842661 PMC8033329

[10] Fetter T Niebel D Braegelmann C Wenzel J. Skin-associated B cells in the pathogenesis of cutaneous autoimmune diseases-implications for therapeutic approaches. Cells (2020) 9(12):2627. doi: 10.3390/cells9122627 33297481 PMC7762338

[11] Nihal M Mikkola D Wood GS. Detection of clonally restricted immunoglobulin heavy chain gene rearrangements in normal and lesional skin: analysis of the B cell component of the skin-associated lymphoid tissue and implications for the molecular diagnosis of cutaneous B cell lymphomas. J Mol Diagn (2000) 2:5–10. doi: 10.1016/S1525-1578(10)60609-5 11272902 PMC1906891

[12] Geherin SA Fintushel SR Lee MH Wilson RP Patel RT Alt C. The skin, a novel niche for recirculating B cells. J Immunol (2012) 188:6027–35. doi: 10.4049/jimmunol.1102639 PMC3370056 22561151

[13] Neeland MR Meeusen EN de Veer MJ. Afferent lymphatic cannulation as a model system to study innate immune responses to infection and vaccination. Vet Immunol Immunopathol (2014) 158:86–97. doi: 10.1016/j.vetimm.2013.01.004 23369582

[14] Geherin SA Gomez D Glabman RA Ruthel G Hamann A Debes GF. Il-10+ Innate-like B cells are part of the skin immune system and require alpha4beta1 integrin to migrate between the peritoneum and inflamed skin. J Immunol (2016) 196:2514–25. doi: 10.4049/jimmunol.1403246 PMC4779667 26851219

[15] Bos JD Zonneveld I Das PK Krieg SR van der Loos CM Kapsenberg ML. The skin immune system (Sis): distribution and immunophenotype of lymphocyte subpopulations in normal human skin. J Invest Dermatol (1987) 88:569–73. doi: 10.1111/1523-1747.ep12470172 3494791

[16] Olszewski WL Grzelak I Ziolkowska A Engeset A. Immune cell traffic from blood through the normal human skin to lymphatics. Clin Dermatol (1995) 13:473–83. doi: 10.1016/0738-081x(95)00087-v 8665458

[17] Genestier L Taillardet M Mondiere P Gheit H Bella C DeFrance T. Tlr agonists selectively promote terminal plasma cell differentiation of B cell subsets specialized in thymus-independent responses. J Immunol (2007) 178:7779–86. doi: 10.4049/jimmunol.178.12.7779 17548615

[18] Ha SA Tsuji M Suzuki K Meek B Yasuda N Kaisho T. Regulation of B1 cell migration by signals through toll-like receptors. J Exp Med (2006) 203:2541–50. doi: 10.1084/jem.20061041 PMC2118139 17060475

[19] Kreuk LS Koch MA Slayden LC Lind NA Chu S Savage HP. B cell receptor and toll-like receptor signaling coordinate to control distinct B-1 responses to both self and the microbiota. Elife (2019) 8:e47015. doi: 10.7554/eLife.47015 31433298 PMC6703855

[20] Saul L Ilieva KM Bax HJ Karagiannis P Correa I Rodriguez-Hernandez I. Igg subclass switching and clonal expansion in cutaneous melanoma and normal skin. Sci Rep (2016) 6:29736. doi: 10.1038/srep29736 27411958 PMC4944184

[21] Okada T Konishi H Ito M Nagura H Asai J. Identification of secretory immunoglobulin a in human sweat and sweat glands. J Invest Dermatol (1988) 90:648–51. doi: 10.1111/1523-1747.ep12560807 3283249

[22] Metze D Jurecka W Gebhart W Schmidt J Mainitz M Niebauer G. Immunohistochemical demonstration of immunoglobulin a in human sebaceous and sweat glands. J Invest Dermatol (1989) 92:13–7. doi: 10.1111/1523-1747.ep13070402 2642508

[23] Metze D Kersten A Jurecka W Gebhart W. Immunoglobulins coat microorganisms of skin surface: A comparative immunohistochemical and ultrastructural study of cutaneous and oral microbial symbionts. J Invest Dermatol (1991) 96:439–45. doi: 10.1111/1523-1747.ep12469908 2007782

[24] Wilson RP McGettigan SE Dang VD Kumar A Cancro MP Nikbakht N. Igm plasma cells reside in healthy skin and accumulate with chronic inflammation. J Invest Dermatol (2019) 139:2477–87. doi: 10.1016/j.jid.2019.05.009 PMC6874734 31152755

[25] Suchanek O Ferdinand JR Tuong ZK Wijeyesinghe S Chandra A Clauder AK. Tissue-resident B cells orchestrate macrophage polarisation and function. Nat Commun (2023) 14:7081. doi: 10.1038/s41467-023-42625-4 37925420 PMC10625551

[26] Naik S Bouladoux N Wilhelm C Molloy MJ Salcedo R Kastenmuller W. Compartmentalized control of skin immunity by resident commensals. Science (2012) 337:1115–9. doi: 10.1126/science.1225152 PMC3513834 22837383

[27] Iwata Y Yoshizaki A Komura K Shimizu K Ogawa F Hara T. Cd19, a response regulator of B lymphocytes, regulates wound healing through hyaluronan-induced tlr4 signaling. Am J Pathol (2009) 175:649–60. doi: 10.2353/ajpath.2009.080355 PMC2716964 19574428

[28] Joo HM He Y Sangster MY. Broad dispersion and lung localization of virus-specific memory B cells induced by influenza pneumonia. Proc Natl Acad Sci U.S.A (2008) 105:3485–90. doi: 10.1073/pnas.0800003105 PMC2265167 18299574

[29] Onodera T Takahashi Y Yokoi Y Ato M Kodama Y Hachimura S. Memory B cells in the lung participate in protective humoral immune responses to pulmonary influenza virus reinfection. Proc Natl Acad Sci U.S.A (2012) 109:2485–90. doi: 10.1073/pnas.1115369109 PMC3289300 22308386

[30] Adachi Y Onodera T Yamada Y Daio R Tsuiji M Inoue T. Distinct germinal center selection at local sites shapes memory B cell response to viral escape. J Exp Med (2015) 212:1709–23. doi: 10.1084/jem.20142284 PMC4577849 26324444

[31] Allie SR Bradley JE Mudunuru U Schultz MD Graf BA Lund FE. The establishment of resident memory B cells in the lung requires local antigen encounter. Nat Immunol (2019) 20:97–108. doi: 10.1038/s41590-018-0260-6 30510223 PMC6392030

[32] Oh JE Song E Moriyama M Wong P Zhang S Jiang R. Intranasal priming induces local lung-resident B cell populations that secrete protective mucosal antiviral iga. Sci Immunol (2021) 6:eabj5129. doi: 10.1126/sciimmunol.abj5129 34890255 PMC8762609

[33] Lee CM Oh JE. Resident memory B cells in barrier tissues. Front Immunol (2022) 13:953088. doi: 10.3389/fimmu.2022.953088 35924234 PMC9341246

[34] Oh JE Iijima N Song E Lu P Klein J Jiang R. Migrant memory B cells secrete luminal antibody in the vagina. Nature (2019) 571:122–6. doi: 10.1038/s41586-019-1285-1 PMC6609483 31189952

[35] Gebhardt T Palendira U Tscharke DC Bedoui S. Tissue-resident memory T cells in tissue homeostasis, persistent infection, and cancer surveillance. Immunol Rev (2018) 283:54–76. doi: 10.1111/imr.12650 29664571

[36] Tokura Y Phadungsaksawasdi P Kurihara K Fujiyama T Honda T. Pathophysiology of skin resident memory T cells. Front Immunol (2020) 11:618897. doi: 10.3389/fimmu.2020.618897 33633737 PMC7901930

[37] Berg EL Yoshino T Rott LS Robinson MK Warnock RA Kishimoto TK. The cutaneous lymphocyte antigen is a skin lymphocyte homing receptor for the vascular lectin endothelial cell-leukocyte adhesion molecule 1. J Exp Med (1991) 174:1461–6. doi: 10.1084/jem.174.6.1461 PMC2119052 1720810

[38] Groves RW Ross E Barker JN Ross JS Camp RD MacDonald DM. Effect of in vivo interleukin-1 on adhesion molecule expression in normal human skin. J Invest Dermatol (1992) 98:384–7. doi: 10.1111/1523-1747.ep12499816 1372029

[39] Yoshino T Okano M Chen HL Tsuchiyama J Kondo E Nishiuchi R. Cutaneous lymphocyte antigen is expressed on memory/effector B cells in the peripheral blood and monocytoid B cells in the lymphoid tissues. Cell Immunol (1999) 197:39–45. doi: 10.1006/cimm.1999.1552 10555994

[40] Rott LS Briskin MJ Butcher EC. Expression of alpha4beta7 and E-selectin ligand by circulating memory B cells: implications for targeted trafficking to mucosal and systemic sites. J Leukoc Biol (2000) 68:807–14. doi: 10.1189/jlb.68.6.807 11129647

[41] Johansson C Ahlstedt I Furubacka S Johnsson E Agace WW Quiding-Jarbrink M. Differential expression of chemokine receptors on human iga+ and igg+ B cells. Clin Exp Immunol (2005) 141:279–87. doi: 10.1111/j.1365-2249.2005.02843.x PMC1809445 15996192

[42] Corcione A Tortolina G Bonecchi R Battilana N Taborelli G Malavasi F. Chemotaxis of human tonsil B lymphocytes to cc chemokine receptor (Ccr) 1, ccr2 and ccr4 ligands is restricted to non-germinal center cells. Int Immunol (2002) 14:883–92. doi: 10.1093/intimm/dxf054 12147625

[43] Kunkel EJ Butcher EC. Plasma-cell homing. Nat Rev Immunol (2003) 3:822–9. doi: 10.1038/nri1203 14523388

[44] Teraki Y Moriya N Shiohara T. Drug-induced expression of intercellular adhesion molecule-1 on lesional keratinocytes in fixed drug eruption. Am J Pathol (1994) 145:550–60. PMC1890340 7915886

[45] Shiohara T Moriya N. Epidermal T cells: their functional role and disease relevance for dermatologists. J Invest Dermatol (1997) 109:271–5. doi: 10.1111/1523-1747.ep12335465 9284089

[46] Teraki Y Shiohara T. Ifn-gamma-producing effector cd8+ T cells and il-10-producing regulatory cd4+ T cells in fixed drug eruption. J Allergy Clin Immunol (2003) 112:609–15. doi: 10.1016/s0091-6749(03)01624-5 13679823

[47] Yuan H Zhou S Liu Z Cong W Fei X Zeng W. Pivotal role of lesional and perilesional T/B lymphocytes in pemphigus pathogenesis. J Invest Dermatol (2017) 137:2362–70. doi: 10.1016/j.jid.2017.05.032 28647348

[48] Bosello S Angelucci C Lama G Alivernini S Proietti G Tolusso B. Characterization of inflammatory cell infiltrate of scleroderma skin: B cells and skin score progression. Arthritis Res Ther (2018) 20:75. doi: 10.1186/s13075-018-1569-0 29669578 PMC5907298

[49] Czarnowicki T Gonzalez J Bonifacio KM Shemer A Xiangyu P Kunjravia N. Diverse activation and differentiation of multiple B-cell subsets in patients with atopic dermatitis but not in patients with psoriasis. J Allergy Clin Immunol (2016) 137:118–29 e5. doi: 10.1016/j.jaci.2015.08.027 26441226

[50] Stanley JR. Autoantibodies against adhesion molecules and structures in blistering skin diseases. J Exp Med (1995) 181:1–4. doi: 10.1084/jem.181.1.1 7806996 PMC2191837

[51] Egami S Watanabe T Fukushima-Nomura A Nomura H Takahashi H Yamagami J. Desmoglein-specific B-cell-targeted single-cell analysis revealing unique gene regulation in patients with pemphigus. J Invest Dermatol (2023) 143(10):1919–28.e16. doi: 10.1016/j.jid.2023.03.1661 36997112

[52] Pollmann R Walter E Schmidt T Waschke J Hertl M Mobs C. Identification of autoreactive B cell subpopulations in peripheral blood of autoimmune patients with pemphigus vulgaris. Front Immunol (2019) 10:1375. doi: 10.3389/fimmu.2019.01375 31258541 PMC6587433

[53] Abrikosova VA Mokrushina YA Ovchinnikova LA Larina EN Terekhov SS Baranova MN. B cell profiling in patients with pemphigus vulgaris. Acta Naturae (2023) 15:13–8. doi: 10.32607/actanaturae.11890 PMC10154782 37153513

[54] Hammers CM Stanley JR. Mechanisms of disease: pemphigus and bullous pemphigoid. Annu Rev Pathol (2016) 11:175–97. doi: 10.1146/annurev-pathol-012615-044313 PMC5560122 26907530

[55] Zhou S Liu Z Yuan H Zhao X Zou Y Zheng J. Autoreactive B cell differentiation in diffuse ectopic lymphoid-like structures of inflamed pemphigus lesions. J Invest Dermatol (2020) 140:309–18 e8. doi: 10.1016/j.jid.2019.07.717 31476317

[56] Kurien BT Scofield RH. Autoantibody determination in the diagnosis of systemic lupus erythematosus. Scand J Immunol (2006) 64:227–35. doi: 10.1111/j.1365-3083.2006.01819.x 16918691

[57] Harrist TJ Mihm MC Jr. The specificity and clinical usefulness of the lupus band test. Arthritis Rheum (1980) 23:479–90. doi: 10.1002/art.1780230411 6989372

[58] Lyons R Narain S Nichols C Satoh M Reeves WH. Effective use of autoantibody tests in the diagnosis of systemic autoimmune disease. Ann N Y Acad Sci (2005) 1050:217–28. doi: 10.1196/annals.1313.023 16014537

[59] Yurasov S Wardemann H Hammersen J Tsuiji M Meffre E Pascual V. Defective B cell tolerance checkpoints in systemic lupus erythematosus. J Exp Med (2005) 201:703–11. doi: 10.1084/jem.20042251 PMC2212839 15738055

[60] Fu Q Zhang X. From blood to tissue: take a deeper look at B cells in lupus. Cell Mol Immunol (2021) 18:2073–4. doi: 10.1038/s41423-021-00713-9 PMC8322426 34117369

[61] Sims GP Ettinger R Shirota Y Yarboro CH Illei GG Lipsky PE. Identification and characterization of circulating human transitional B cells. Blood (2005) 105:4390–8. doi: 10.1182/blood-2004-11-4284 PMC1895038 15701725

[62] Wing E Sutherland C Miles K Gray D Goodyear CS Otto TD. Double-negative-2 B cells are the major synovial plasma cell precursor in rheumatoid arthritis. Front Immunol (2023) 14:1241474. doi: 10.3389/fimmu.2023.1241474 37638026 PMC10450142

[63] Hussein MR Aboulhagag NM Atta HS Atta SM. Evaluation of the profile of the immune cell infiltrate in lichen planus, discoid lupus erythematosus, and chronic dermatitis. Pathology (2008) 40:682–93. doi: 10.1080/00313020802320739 18728929

[64] Thorpe RB Gray A Kumar KR Susa JS Chong BF. Site-specific analysis of inflammatory markers in discoid lupus erythematosus skin. ScientificWorldJournal (2014) 2014:925805. doi: 10.1155/2014/925805 24744689 PMC3972874

[65] Massone C Kodama K Salmhofer W Abe R Shimizu H Parodi A. Lupus erythematosus panniculitis (Lupus profundus): clinical, histopathological, and molecular analysis of nine cases. J Cutan Pathol (2005) 32:396–404. doi: 10.1111/j.0303-6987.2005.00351.x 15953372

[66] Kogame T Yamashita R Hirata M Kataoka TR Kamido H Ueshima C. Analysis of possible structures of inducible skin-associated lymphoid tissue in lupus erythematosus profundus. J Dermatol (2018) 45:1117–21. doi: 10.1111/1346-8138.14498 29897143

[67] Dey-Rao R Sinha AA. In silico analyses of skin and peripheral blood transcriptional data in cutaneous lupus reveals ccr2-a novel potential therapeutic target. Front Immunol (2019) 10:640. doi: 10.3389/fimmu.2019.00640 30984198 PMC6450170

[68] Wenzel J Landmann A Vorwerk G Kuhn A. High expression of B lymphocyte stimulator in lesional keratinocytes of patients with cutaneous lupus erythematosus. Exp Dermatol (2018) 27:95–7. doi: 10.1111/exd.13419 28833566

[69] Weidinger S Beck LA Bieber T Kabashima K Irvine AD. Atopic dermatitis. Nat Rev Dis Primers (2018) 4:1. doi: 10.1038/s41572-018-0001-z 29930242

[70] Tang TS Bieber T Williams HC. Does “Autoreactivity” Play a role in atopic dermatitis? J Allergy Clin Immunol (2012) 129:1209–15 e2. doi: 10.1016/j.jaci.2012.02.002 22409986

[71] Hradetzky S Werfel T Rosner LM. Autoallergy in atopic dermatitis. Allergo J Int (2015) 24:16–22. doi: 10.1007/s40629-015-0037-5 26120543 PMC4479480

[72] De Bruyn Carlier T Badloe FMS Ring J Gutermuth J Kortekaas Krohn I. Autoreactive T cells and their role in atopic dermatitis. J Autoimmun (2021) 120:102634. doi: 10.1016/j.jaut.2021.102634 33892348

[73] Tokura Y Hayano S. Subtypes of atopic dermatitis: from phenotype to endotype. Allergol Int (2022) 71:14–24. doi: 10.1016/j.alit.2021.07.003 34344611

[74] Mobus L Rodriguez E Harder I Boraczynski N Szymczak S Hubenthal M. Blood transcriptome profiling identifies 2 candidate endotypes of atopic dermatitis. J Allergy Clin Immunol (2022) 150:385–95. doi: 10.1016/j.jaci.2022.02.001 35182548

[75] Yanaba K Kamata M Asano Y Tada Y Sugaya M Kadono T. Cd19 expression in B cells regulates atopic dermatitis in a mouse model. Am J Pathol (2013) 182:2214–22. doi: 10.1016/j.ajpath.2013.02.042 PMC3668019 23583649

[76] Duan L Liu D Chen H Mintz MA Chou MY Kotov DI. Follicular dendritic cells restrict interleukin-4 availability in germinal centers and foster memory B cell generation. Immunity (2021) 54:2256–72.e6. doi: 10.1016/j.immuni.2021.08.028 34555336 PMC8516727

[77] Pignarre A Chatonnet F Caron G Haas M Desmots F Fest T. Plasmablasts derive from cd23- activated B cells after the extinction of il-4/stat6 signaling and irf4 induction. Blood (2021) 137:1166–80. doi: 10.1182/blood.2020005083 33150420

[78] Santamaria K Desmots F Leonard S Caron G Haas M Delaloy C. Committed human cd23-negative light-zone germinal center B cells delineate transcriptional program supporting plasma cell differentiation. Front Immunol (2021) 12:744573. doi: 10.3389/fimmu.2021.744573 34925321 PMC8674954

[79] Lee CE Yoon SR Pyun KH. Interleukin-4 signals regulating cd23 gene expression in human B cells: protein kinase C-independent signaling pathways. Cell Immunol (1993) 146:171–85. doi: 10.1006/cimm.1993.1015 8425225

[80] Mauri C. Regulation of immunity and autoimmunity by B cells. Curr Opin Immunol (2010) 22:761–7. doi: 10.1016/j.coi.2010.10.009 21087847

[81] Candando KM Lykken JM Tedder TF. B10 cell regulation of health and disease. Immunol Rev (2014) 259:259–72. doi: 10.1111/imr.12176 PMC4049540 24712471

[82] Correale J Farez M Razzitte G. Helminth infections associated with multiple sclerosis induce regulatory B cells. Ann Neurol (2008) 64:187–99. doi: 10.1002/ana.21438 18655096

[83] Blair PA Norena LY Flores-Borja F Rawlings DJ Isenberg DA Ehrenstein MR. Cd19(+)Cd24(Hi)Cd38(Hi) B cells exhibit regulatory capacity in healthy individuals but are functionally impaired in systemic lupus erythematosus patients. Immunity (2010) 32:129–40. doi: 10.1016/j.immuni.2009.11.009 20079667

[84] Iwata Y Matsushita T Horikawa M Dilillo DJ Yanaba K Venturi GM. Characterization of a rare il-10-competent B-cell subset in humans that parallels mouse regulatory B10 cells. Blood (2011) 117:530–41. doi: 10.1182/blood-2010-07-294249 PMC3031478 20962324

[85] Ponte P Lopes MJ. Apparent safe use of single dose rituximab for recalcitrant atopic dermatitis in the first trimester of a twin pregnancy. J Am Acad Dermatol (2010) 63:355–6. doi: 10.1016/j.jaad.2009.05.015 20633812

[86] Schmidt E Seitz CS Benoit S Brocker EB Goebeler M. Rituximab in autoimmune bullous diseases: mixed responses and adverse effects. Br J Dermatol (2007) 156:352–6. doi: 10.1111/j.1365-2133.2006.07646.x 17223877

[87] Kersh AE Feldman RJ. Autoimmune sequelae following rituximab therapy: A review of the literature and potential immunologic mechanisms. J Clin Rheumatol (2018) 24:427–35. doi: 10.1097/RHU.0000000000000756 29561469

[88] Dass S Vital EM Emery P. Development of psoriasis after B cell depletion with rituximab. Arthritis Rheum (2007) 56:2715–8. doi: 10.1002/art.22811 17665440

[89] Mielke F Schneider-Obermeyer J Dorner T. Onset of psoriasis with psoriatic arthropathy during rituximab treatment of non-hodgkin lymphoma. Ann Rheum Dis (2008) 67:1056–7. doi: 10.1136/ard.2007.080929 18556453

[90] Suga H Sato S. Il-10-producing regulatory B cells in skin diseases. J Cutan Immunol All (2019) 2:68–74. doi: 10.1002/cia2.12059

[91] Yang X Yang J Chu Y Xue Y Xuan D Zheng S. T follicular helper cells and regulatory B cells dynamics in systemic lupus erythematosus. PloS One (2014) 9:e88441. doi: 10.1371/journal.pone.0088441 24551101 PMC3925141

[92] Kabuto M Fujimoto N Tanaka T. Increase of interleukin-10-producing B cells associated with long-term remission after I.V. Immunoglobulin treatment for pemphigus. J Dermatol (2016) 43:815–8. doi: 10.1111/1346-8138.13295 26871259

[93] Kabuto M Fujimoto N Takahashi T Tanaka T. Decreased level of interleukin-10-producing B cells in patients with pemphigus but not in patients with pemphigoid. Br J Dermatol (2017) 176:1204–12. doi: 10.1111/bjd.15113 27716906

[94] Colliou N Picard D Caillot F Calbo S Le Corre S Lim A. Long-term remissions of severe pemphigus after rituximab therapy are associated with prolonged failure of desmoglein B cell response. Sci Transl Med (2013) 5:175ra30. doi: 10.1126/scitranslmed.3005166 23467561

[95] Zhu HQ Xu RC Chen YY Yuan HJ Cao H Zhao XQ. Impaired function of cd19(+) cd24(Hi) cd38(Hi) regulatory B cells in patients with pemphigus. Br J Dermatol (2015) 172:101–10. doi: 10.1111/bjd.13192 24935080

[96] Li J Shen C Liu Y Li Y Sun L Jiao L. Impaired function of cd5+Cd19+Cd1dhi B10 cells on ige secretion in an atopic dermatitis-like mouse model. PloS One (2015) 10:e0132173. doi: 10.1371/journal.pone.0132173 26244559 PMC4526574

[97] Yoshihara Y Ishiuji Y Yoshizaki A Kurita M Hayashi M Ishiji T. Il-10-producing regulatory B cells are decreased in patients with atopic dermatitis. J Invest Dermatol (2019) 139:475–8. doi: 10.1016/j.jid.2018.08.016 30236612

[98] Jiang J Yan S Zhou X Zhou J Bai X Tan Q. Crosstalk between circulating follicular T helper cells and regulatory B cells in children with extrinsic atopic dermatitis. Front Immunol (2021) 12:785549. doi: 10.3389/fimmu.2021.785549 34917093 PMC8669722

[99] Matsushita T Hamaguchi Y Hasegawa M Takehara K Fujimoto M. Decreased levels of regulatory B cells in patients with systemic sclerosis: association with autoantibody production and disease activity. Rheumatol (Oxford) (2016) 55:263–7. doi: 10.1093/rheumatology/kev331 26350483

[100] Matsushita T Kobayashi T Mizumaki K Kano M Sawada T Tennichi M. Baff inhibition attenuates fibrosis in scleroderma by modulating the regulatory and effector B cell balance. Sci Adv (2018) 4:eaas9944. doi: 10.1126/sciadv.aas9944 30009261 PMC6040844

[101] Nagel A Hertl M Eming R. B-cell-directed therapy for inflammatory skin diseases. J Invest Dermatol (2009) 129:289–301. doi: 10.1038/jid.2008.192 19148218

[102] Lee DSW Rojas OL Gommerman JL. B cell depletion therapies in autoimmune disease: advances and mechanistic insights. Nat Rev Drug Discovery (2021) 20:179–99. doi: 10.1038/s41573-020-00092-2 PMC7737718 33324003

[103] Uchida J Hamaguchi Y Oliver JA Ravetch JV Poe JC Haas KM. The innate mononuclear phagocyte network depletes B lymphocytes through fc receptor-dependent mechanisms during anti-cd20 antibody immunotherapy. J Exp Med (2004) 199:1659–69. doi: 10.1084/jem.20040119 PMC2212805 15210744

[104] Joly P Mouquet H Roujeau JC D’Incan M Gilbert D Jacquot S. A single cycle of rituximab for the treatment of severe pemphigus. N Engl J Med (2007) 357:545–52. doi: 10.1056/NEJMoa067752 17687130

[105] Vinay K Kanwar AJ Mittal A Dogra S Minz RW Hashimoto T. Intralesional rituximab in the treatment of refractory oral pemphigus vulgaris. JAMA Dermatol (2015) 151:878–82. doi: 10.1001/jamadermatol.2014.3674 25536513

[106] Iraji F Danesh F Faghihi G Siadat A Mokhtari F Talakoob M. Comparison between the efficacy of intralesional rituximab versus intralesional triamcinolone in the treatment refractory pemphigus vulgaris lesions: A randomized clinical trial. Int Immunopharmacol (2019) 73:94–7. doi: 10.1016/j.intimp.2019.04.031 31082727

[107] Edwards JC Szczepanski L Szechinski J Filipowicz-Sosnowska A Emery P Close DR. Efficacy of B-cell-targeted therapy with rituximab in patients with rheumatoid arthritis. N Engl J Med (2004) 350:2572–81. doi: 10.1056/NEJMoa032534 15201414

[108] Cohen SB Emery P Greenwald MW Dougados M Furie RA Genovese MC. Rituximab for rheumatoid arthritis refractory to anti-tumor necrosis factor therapy: results of a multicenter, randomized, double-blind, placebo-controlled, phase iii trial evaluating primary efficacy and safety at twenty-four weeks. Arthritis Rheum (2006) 54:2793–806. doi: 10.1002/art.22025 16947627

[109] Vital EM Wittmann M Edward S Md Yusof MY MacIver H Pease CT. Brief report: responses to rituximab suggest B cell-independent inflammation in cutaneous systemic lupus erythematosus. Arthritis Rheumatol (2015) 67:1586–91. doi: 10.1002/art.39085 25707733

[110] Quelhas da Costa R Aguirre-Alastuey ME Isenberg DA Saracino AM. Assessment of response to B-cell depletion using rituximab in cutaneous lupus erythematosus. JAMA Dermatol (2018) 154:1432–40. doi: 10.1001/jamadermatol.2018.3793 PMC6583321 30383114

[111] Raderer M Jager G Brugger S Puspok A Fiebiger W Drach J. Rituximab for treatment of advanced extranodal marginal zone B cell lymphoma of the mucosa-associated lymphoid tissue lymphoma. Oncology (2003) 65:306–10. doi: 10.1159/000074641 14707449

[112] Simon D Hosli S Kostylina G Yawalkar N Simon HU. Anti-cd20 (Rituximab) treatment improves atopic eczema. J Allergy Clin Immunol (2008) 121:122–8. doi: 10.1016/j.jaci.2007.11.016 18206507

[113] Liu D Xu H Shih C Wan Z Ma X Ma W. T-B-cell entanglement and icosl-driven feed-forward regulation of germinal centre reaction. Nature (2015) 517:214–8. doi: 10.1038/nature13803 25317561

[114] Navarra SV Guzman RM Gallacher AE Hall S Levy RA Jimenez RE. Efficacy and safety of belimumab in patients with active systemic lupus erythematosus: A randomised, placebo-controlled, phase 3 trial. Lancet (2011) 377:721–31. doi: 10.1016/S0140-6736(10)61354-2 21296403

[115] Stohl W Metyas S Tan SM Cheema GS Oamar B Xu D. B lymphocyte stimulator overexpression in patients with systemic lupus erythematosus: longitudinal observations. Arthritis Rheum (2003) 48:3475–86. doi: 10.1002/art.11354 14673998

[116] Chong BF Tseng LC Kim A Miller RT Yancey KB Hosler GA. Differential expression of baff and its receptors in discoid lupus erythematosus patients. J Dermatol Sci (2014) 73:216–24. doi: 10.1016/j.jdermsci.2013.11.007 PMC3946198 24315762

[117] Vashisht P Borghoff K O’Dell JR Hearth-Holmes M. Belimumab for the treatment of recalcitrant cutaneous lupus. Lupus (2017) 26:857–64. doi: 10.1177/0961203316682097 28121495

[118] Murphy G Isenberg DA. New therapies for systemic lupus erythematosus - past imperfect, future tense. Nat Rev Rheumatol (2019) 15:403–12. doi: 10.1038/s41584-019-0235-5 31165780

[119] Dorner T Posch MG Li Y Petricoul O Cabanski M Milojevic JM. Treatment of primary sjogren’s syndrome with ianalumab (Vay736) targeting B cells by baff receptor blockade coupled with enhanced, antibody-dependent cellular cytotoxicity. Ann Rheum Dis (2019) 78:641–7. doi: 10.1136/annrheumdis-2018-214720 30826774

[120] Abulikemu K Hu F Liang J Kang X. Targeting therapy in pemphigus: where are we now and where are we going? Heliyon (2023) 9:e16679. doi: 10.1016/j.heliyon.2023.e16679 37292301 PMC10245244

[121] Patsatsi A Murrell DF. Bruton tyrosine kinase inhibition and its role as an emerging treatment in pemphigus. Front Med (Lausanne) (2021) 8:708071. doi: 10.3389/fmed.2021.708071 34447768 PMC8382970

[122] Lee A Sandhu S Imlay-Gillespie L Mulligan S Shumack S. Successful use of bruton’s kinase inhibitor, ibrutinib, to control paraneoplastic pemphigus in a patient with paraneoplastic autoimmune multiorgan syndrome and chronic lymphocytic leukaemia. Australas J Dermatol (2017) 58:e240–e2. doi: 10.1111/ajd.12615 28295171

[123] Smith PF Krishnarajah J Nunn PA Hill RJ Karr D Tam D. A phase I trial of prn1008, a novel reversible covalent inhibitor of bruton’s tyrosine kinase, in healthy volunteers. Br J Clin Pharmacol (2017) 83:2367–76. doi: 10.1111/bcp.13351 PMC5651318 28636208

[124] Edner NM Carlesso G Rush JS Walker LSK. Targeting co-stimulatory molecules in autoimmune disease. Nat Rev Drug Discovery (2020) 19:860–83. doi: 10.1038/s41573-020-0081-9 32939077

[125] Lai JH Luo SF Ho LJ. Targeting the cd40-cd154 signaling pathway for treatment of autoimmune arthritis. Cells (2019) 8(8):927. doi: 10.3390/cells8080927 31426619 PMC6721639

[126] Armitage RJ Fanslow WC Strockbine L Sato TA Clifford KN Macduff BM. Molecular and biological characterization of a murine ligand for cd40. Nature (1992) 357:80–2. doi: 10.1038/357080a0 1374165

[127] Tokunaga M Fujii K Saito K Nakayamada S Tsujimura S Nawata M. Down-regulation of cd40 and cd80 on B cells in patients with life-threatening systemic lupus erythematosus after successful treatment with rituximab. Rheumatol (Oxford) (2005) 44:176–82. doi: 10.1093/rheumatology/keh443 15494350

[128] Tokunaga M Saito K Kawabata D Imura Y Fujii T Nakayamada S. Efficacy of rituximab (Anti-cd20) for refractory systemic lupus erythematosus involving the central nervous system. Ann Rheum Dis (2007) 66:470–5. doi: 10.1136/ard.2006.057885 PMC1856059 17107983

[129] Huang W Sinha J Newman J Reddy B Budhai L Furie R. The effect of anti-cd40 ligand antibody on B cells in human systemic lupus erythematosus. Arthritis Rheum (2002) 46:1554–62. doi: 10.1002/art.10273 12115186

[130] Xie JH Yamniuk AP Borowski V Kuhn R Susulic V Rex-Rabe S. Engineering of a novel anti-cd40l domain antibody for treatment of autoimmune diseases. J Immunol (2014) 192:4083–92. doi: 10.4049/jimmunol.1303239 24670803

[131] Shock A Burkly L Wakefield I Peters C Garber E Ferrant J. Cdp7657, an anti-cd40l antibody lacking an fc domain, inhibits cd40l-dependent immune responses without thrombotic complications: an in vivo study. Arthritis Res Ther (2015) 17:234. doi: 10.1186/s13075-015-0757-4 26335795 PMC4558773

[132] Bhoj VG Arhontoulis D Wertheim G Capobianchi J Callahan CA Ellebrecht CT. Persistence of long-lived plasma cells and humoral immunity in individuals responding to cd19-directed car T-cell therapy. Blood (2016) 128:360–70. doi: 10.1182/blood-2016-01-694356 PMC4957161 27166358

[133] Bach PB Giralt SA Saltz LB. Fda approval of tisagenlecleucel: promise and complexities of a $475 000 cancer drug. JAMA (2017) 318:1861–2. doi: 10.1001/jama.2017.15218 28975266

[134] Ellebrecht CT Bhoj VG Nace A Choi EJ Mao X Cho MJ. Reengineering chimeric antigen receptor T cells for targeted therapy of autoimmune disease. Science (2016) 353:179–84. doi: 10.1126/science.aaf6756 PMC5343513 27365313

[135] Lee J Lundgren DK Mao X Manfredo-Vieira S Nunez-Cruz S Williams EF. Antigen-specific B cell depletion for precision therapy of mucosal pemphigus vulgaris. J Clin Invest (2020) 130:6317–24. doi: 10.1172/JCI138416 PMC7685721 32817591

[136] Ward DE Fay BL Adejuwon A Han H Ma Z. Chimeric antigen receptors based on low affinity mutants of fcepsilonri re-direct T cell specificity to cells expressing membrane ige. Front Immunol (2018) 9:2231. doi: 10.3389/fimmu.2018.02231 30364107 PMC6191488

[137] Stasi R Stipa E Del Poeta G Amadori S Newland AC Provan D. Long-term observation of patients with anti-neutrophil cytoplasmic antibody-associated vasculitis treated with rituximab. Rheumatol (Oxford) (2006) 45:1432–6. doi: 10.1093/rheumatology/kel098 16632482

[138] Kyriakidis I Mantadakis E Stiakaki E Groll AH Tragiannidis A. Infectious complications of targeted therapies in children with leukemias and lymphomas. Cancers (Basel) (2022) 14(20):5022. doi: 10.3390/cancers14205022 36291806 PMC9599435

[139] Kornek B Leutmezer F Rommer PS Koblischke M Schneider L Haslacher H. B cell depletion and sars-cov-2 vaccine responses in neuroimmunologic patients. Ann Neurol (2022) 91:342–52. doi: 10.1002/ana.26309 PMC9011809 35067959

[140] Oren S Mandelboim M Braun-Moscovici Y Paran D Ablin J Litinsky I. Vaccination against influenza in patients with rheumatoid arthritis: the effect of rituximab on the humoral response. Ann Rheum Dis (2008) 67:937–41. doi: 10.1136/ard.2007.077461 17981914

[141] Spelman T Forsberg L McKay K Glaser A Hillert J. Increased rate of hospitalisation for covid-19 among rituximab-treated multiple sclerosis patients: A study of the swedish multiple sclerosis registry. Mult Scler (2022) 28:1051–9. doi: 10.1177/13524585211026272 34212816

[142] Tolf A Wiberg A Muller M Nazir FH Pavlovic I Lauren I. Factors associated with serological response to sars-cov-2 vaccination in patients with multiple sclerosis treated with rituximab. JAMA Netw Open (2022) 5:e2211497. doi: 10.1001/jamanetworkopen.2022.11497 35544139 PMC9096596

[143] Nissimov N Hajiyeva Z Torke S Grondey K Bruck W Hausser-Kinzel S. B cells reappear less mature and more activated after their anti-cd20-mediated depletion in multiple sclerosis. Proc Natl Acad Sci U.S.A (2020) 117:25690–9. doi: 10.1073/pnas.2012249117 PMC7568262 32999069

[144] Mahevas M Michel M Weill JC Reynaud CA. Long-lived plasma cells in autoimmunity: lessons from B-cell depleting therapy. Front Immunol (2013) 4:494. doi: 10.3389/fimmu.2013.00494 24409184 PMC3873528

[145] Hoyer BF Moser K Hauser AE Peddinghaus A Voigt C Eilat D. Short-lived plasmablasts and long-lived plasma cells contribute to chronic humoral autoimmunity in nzb/W mice. J Exp Med (2004) 199:1577–84. doi: 10.1084/jem.20040168 PMC2211779 15173206

[146] Chakma CR Good-Jacobson KL. Requirements of il-4 during the generation of B cell memory. J Immunol (2023) 210:1853–60. doi: 10.4049/jimmunol.2200922 37276051

[147] Gaya M Barral P Burbage M Aggarwal S Montaner B Warren Navia A. Initiation of antiviral B cell immunity relies on innate signals from spatially positioned nkt cells. Cell (2018) 172:517–33.e20. doi: 10.1016/j.cell.2017.11.036 29249358 PMC5786505

[148] Miyauchi K Adachi Y Tonouchi K Yajima T Harada Y Fukuyama H. Influenza virus infection expands the breadth of antibody responses through il-4 signalling in B cells. Nat Commun (2021) 12:3789. doi: 10.1038/s41467-021-24090-z 34145279 PMC8213721

[149] Shehata L Thouvenel CD Hondowicz BD Pew LA Rawlings DJ Choi J. Il-4 downregulates bcl6 to promote memory B cell selection in germinal centers. bioRxiv (2023). doi: 10.1101/2023.01.26.525749 PMC11104266 38513666

[150] Goodnow CC Crosbie J Jorgensen H Brink RA Basten A. Induction of self-tolerance in mature peripheral B lymphocytes. Nature (1989) 342:385–91. doi: 10.1038/342385a0 2586609

[151] Noelle R Krammer PH Ohara J Uhr JW Vitetta ES. Increased expression of ia antigens on resting B cells: an additional role for B-cell growth factor. Proc Natl Acad Sci U.S.A (1984) 81:6149–53. doi: 10.1073/pnas.81.19.6149 PMC391877 6435125

[152] DeFrance T Aubry JP Rousset F Vanbervliet B Bonnefoy JY Arai N. Human recombinant interleukin 4 induces fc epsilon receptors (Cd23) on normal human B lymphocytes. J Exp Med (1987) 165:1459–67. doi: 10.1084/jem.165.6.1459 PMC2188364 2953844

[153] Choe J Kim HS Armitage RJ Choi YS. The functional role of B cell antigen receptor stimulation and il-4 in the generation of human memory B cells from germinal center B cells. J Immunol (1997) 159:3757–66. doi: 10.4049/jimmunol.159.8.3757 9378962

[154] Zhang X Li L Jung J Xiang S Hollmann C Choi YS. The distinct roles of T cell-derived cytokines and a novel follicular dendritic cell-signaling molecule 8d6 in germinal center-B cell differentiation. J Immunol (2001) 167:49–56. doi: 10.4049/jimmunol.167.1.49 11418631

[155] Meli AP Fontes G Leung Soo C King IL. T follicular helper cell-derived il-4 is required for ige production during intestinal helminth infection. J Immunol (2017) 199:244–52. doi: 10.4049/jimmunol.1700141 28533444

[156] Kubo M. The role of il-4 derived from follicular helper T (Tfh) cells and type 2 helper T (Th2) cells. Int Immunol (2021) 33:717–22. doi: 10.1093/intimm/dxab080 34628505

[157] Davari DR Nieman EL McShane DB Morrell DS. Current perspectives on the systemic management of atopic dermatitis. J Asthma Allergy (2021) 14:595–607. doi: 10.2147/JAA.S287638 34103945 PMC8179820

[158] Harb H Chatila TA. Mechanisms of dupilumab. Clin Exp Allergy (2020) 50:5–14. doi: 10.1111/cea.13491 31505066 PMC6930967

[159] Simpson EL Bieber T Guttman-Yassky E Beck LA Blauvelt A Cork MJ. Two phase 3 trials of dupilumab versus placebo in atopic dermatitis. N Engl J Med (2016) 375:2335–48. doi: 10.1056/NEJMoa1610020 27690741

[160] Hansel K Patruno C Antonelli E Dal Bello G Napolitano M Fabbrocini G. Dupilumab in adolescents with moderate to severe atopic dermatitis: A 32-week real-world experience during the covid-19 pandemic. Clin Exp Dermatol (2022) 47:165–7. doi: 10.1111/ced.14862 PMC8444687 34309892

[161] Blauvelt A de Bruin-Weller M Gooderham M Cather JC Weisman J Pariser D. Long-term management of moderate-to-severe atopic dermatitis with dupilumab and concomitant topical corticosteroids (Liberty ad chronos): A 1-year, randomised, double-blinded, placebo-controlled, phase 3 trial. Lancet (2017) 389:2287–303. doi: 10.1016/S0140-6736(17)31191-1 28478972

[162] Yim HJ Jean T Ong PY. Recent advances in immunomodulators for atopic dermatitis. Curr Opin Pediatr (2023) 35(6):671–9. doi: 10.1097/MOP.0000000000001279 37522635

[163] Mountz JD Gao M Ponder DM Liu S Sun CW Alduraibi F. Il-4 receptor blockade is a global repressor of naive B cell development and responses in a dupilumab-treated patient. Clin Immunol (2022) 244:109130. doi: 10.1016/j.clim.2022.109130 36189576 PMC9741950

